# Early-onset group B streptococcal disease in African countries and maternal vaccination strategies

**DOI:** 10.3389/fpubh.2023.1214844

**Published:** 2023-06-29

**Authors:** Ziyaad Dangor, Anna C. Seale, Vuyelwa Baba, Gaurav Kwatra

**Affiliations:** ^1^South African Medical Research Council, Vaccines and Infectious Diseases Analytics Unit, University of the Witwatersrand, Johannesburg, South Africa; ^2^Bill and Melinda Gates Foundation, Seattle, WA, United States; ^3^London School of Hygiene and Tropical Medicine, London, United Kingdom; ^4^Warwick Medical School, University of Warwick, Coventry, United Kingdom; ^5^Department of Obstetrics and Gynaecology, University of the Witwatersrand, Johannesburg, South Africa; ^6^Department of Clinical Microbiology, Christian Medical College, Vellore, India

**Keywords:** Group B Streptococcus, *Streptococcus agalactiae*, early-onset disease, neonatal sepsis, perinatal infections

## Abstract

Invasive group B streptococcal (GBS) disease is the commonest perinatally-acquired bacterial infection in newborns; the burden is higher in African countries where intrapartum antibiotic prophylaxis strategies are not feasible. In sub-Saharan Africa, almost one in four newborns with GBS early-onset disease will demise, and one in ten survivors have moderate or severe neurodevelopmental impairment. A maternal GBS vaccine to prevent invasive GBS disease in infancy is a pragmatic and cost-effective preventative strategy for Africa. Hexavalent polysaccharide protein conjugate and Alpha family surface protein vaccines are undergoing phase II clinical trials. Vaccine licensure may be facilitated by demonstrating safety and immunological correlates/thresholds suggestive of protection against invasive GBS disease. This will then be followed by phase IV effectiveness studies to assess the burden of GBS vaccine preventable disease, including the effect on all-cause neonatal infections, neonatal deaths and stillbirths.

## Introduction

Progress to reduce under five mortality rates by 2030, as part of the sustainable developmental goals, has been modest for many African countries (1), and children living in sub-Saharan Africa are ten-fold more likely to die in the first month after birth compared to high-income countries (1, 2). Many of the deaths in this age group are at and/or around birth; in 2021, of the five-million deaths in children less than 5 years of age, 46% occurred in the neonatal period (1, 2). In this neonatal period, the leading causes of death are from preterm related complications (17%), intrapartum related events (11%) and pneumonia, sepsis or meningitis (8%) (2).

In terms of specific infectious causes, *Streptococcus agalactiae*, commonly referred to as Group B Streptococcus (GBS), is the commonest reported cause of early-onset disease (EOD; i.e.: disease in the first 6 days after birth) in neonates (3–7). However, adverse perinatal outcomes are not limited to EOD in neonates, and late-onset disease (LOD) also occurs mostly within the neonatal period. Whilst considerable mortality is associated with EOD and LOD, survivors may also develop moderate or severe neurodevelopmental impairment (NDI) (8–10). Prior to birth, there may be miscarriages and stillbirth from invasive GBS disease *in-utero* (11, 12), and GBS colonisation has been associated with preterm labour (13, 14). Pregnant women may also develop GBS sepsis (15).

## Burden of disease in Africa

Worldwide, based on maternal GBS colonization, and using Bayesian modelling, there were an estimated 394,000 cases of invasive GBS disease (EOD and LOD) and 58,300 (95%CI: 26,500–125,800) deaths in early infancy (8). The majority of cases of invasive GBS disease, 231,800 (95%CI: 114,100–455,000) cases, were EOD. The burden of disease and death from GBS EOD is high in African countries, with 90,800 (95%CI: 43,000–186,600) of all EOD cases in sub-Saharan Africa (8) and the incidence of EOD almost double the worldwide estimate (16). Consequently, almost half of all early-onset GBS deaths occur in sub-Saharan Africa, where the case fatality is also high (23%, vs. 6% in developed countries) (8). Factors driving the incidence of EOD in African countries include higher prevalence of maternal GBS colonization, and limited or no intrapartum antibiotic prophylaxis (IAP). IAP is commonly given in high-income countries, based on either clinical risk-based screening or microbiological screening to prevent EOD (16, 17). It should also be noted that there are data gaps in Africa, with less than a quarter of African countries contributing data to estimates (8, 16).

The adverse consequences of EOD GBS disease in neonates are not limited to disease and death. There may also be neurodevelopmental impairment (NDI). In high-income countries, survivors of invasive GBS disease (sepsis and/or meningitis) compared to matched controls have a two-fold increased risk of moderate or severe NDI by 10 years of age (9). There is a paucity of long-term outcome data from Africa, but in a recent multi-country matched cohort study undertaken in South Africa, India, Mozambique, Kenya, and Argentina, 38% (8.8% were moderate or severe) of survivors (*n* = 138) of invasive GBS disease had NDI compared to 22% of non-GBS (*n* = 390) children (10). Specifically, survivors of GBS EOD commonly developed NDI (10, 18).

In addition to this burden, there is also a substantial burden from GBS causing stillbirth from infections *in-utero*, with 20,300 (95%CI: 9,000–40,500) out of 46,200 (95%CI: 20,300–111,300) GBS stillbirths in 2020 in sub-Saharan Africa (8). Furthermore, babies born with intrapartum hypoxia from invasive GBS disease may present with neonatal encephalopathy (19, 20).

Maternal GBS colonization increases the risk of preterm labour (13), although data are limited; of the 15 million preterm births that occurred globally in 2020, approximately 518,100 (95%CI: 36,900–1,142,300) were estimated to be associated with GBS, most of which were from sub-Saharan Africa (8). Prematurely born infants also have an increased risk of acquiring invasive GBS disease (21); and being premature and acquiring invasive GBS disease was independenty associated with death and NDI in survivors (18, 22–24).

The acute healthcare cost and impact on the family of a neonate having a sepsis and/or meningitis is important. In a recent study from South Africa (a third of participants had EOD), the mean household and hospital cost of having a child with culture-confirmed GBS, E-coli or Staphyloccal infection was 52 and 684 international dollars, respectively (25). Average hospitalization cost for confirmed sepsis ranged from $55 to $129 in most high-income countries (26). Extra care for a child with invasive GBS disease is also required, and if the child has NDI this will be long-term. In cases of infant deaths from GBS EOD, parents of infants experience grief and express frustrations over the death of the child, and fears of future pregnancy (27).

## Microbiology and pathogenesis of EOD

*Streptococcus agalactiae* is an encepsulated Gram positive coccus exclusively in the “B” Lancefield grouping of *Streptococci*. Approximately 18% (95%CI: 17–19%) of pregnant women are gastrointestinal and/ or genitourinary tract colonised with GBS globally (28); colonization rates are reported to be higher (22–25%) in Southern Africa (28–30), and approximately half of pregnant women are colonized at some point during their pregnancy (31). Of the 20 million pregnant women that had rectovaginal colonization in 2020, 6 (30%) million were living in sub-Saharan Africa (8).

There are ten GBS serotypes (Ia, Ib, II-X), serotype III being the commonest cause of invasive disease followed by Ia and V (16). Geographical differences in the prevalence of serotypes have been reported, and in Africa, serotype III is the commonest for EOD (16); most of the hypervirulent clonal complex 17 is serotype III (32).

There are several risk factors for EOD, GBS colonisation of the pregnant women is the only unequivocal risk factor (33). Other risk factors for EOD include prolonged rupture of membranes (>18 h; PROM), maternal fever during labor, premature labour, previous infant with invasive GBS disease, maternal GBS bacteriuria, chorioamnionitis, absence of screening for GBS colonisation, unavailability of intrapartum antibiotic prophylaxix (IAP), unavailability of a skilled birth attendant (8, 22, 34).

**Figure 1:**
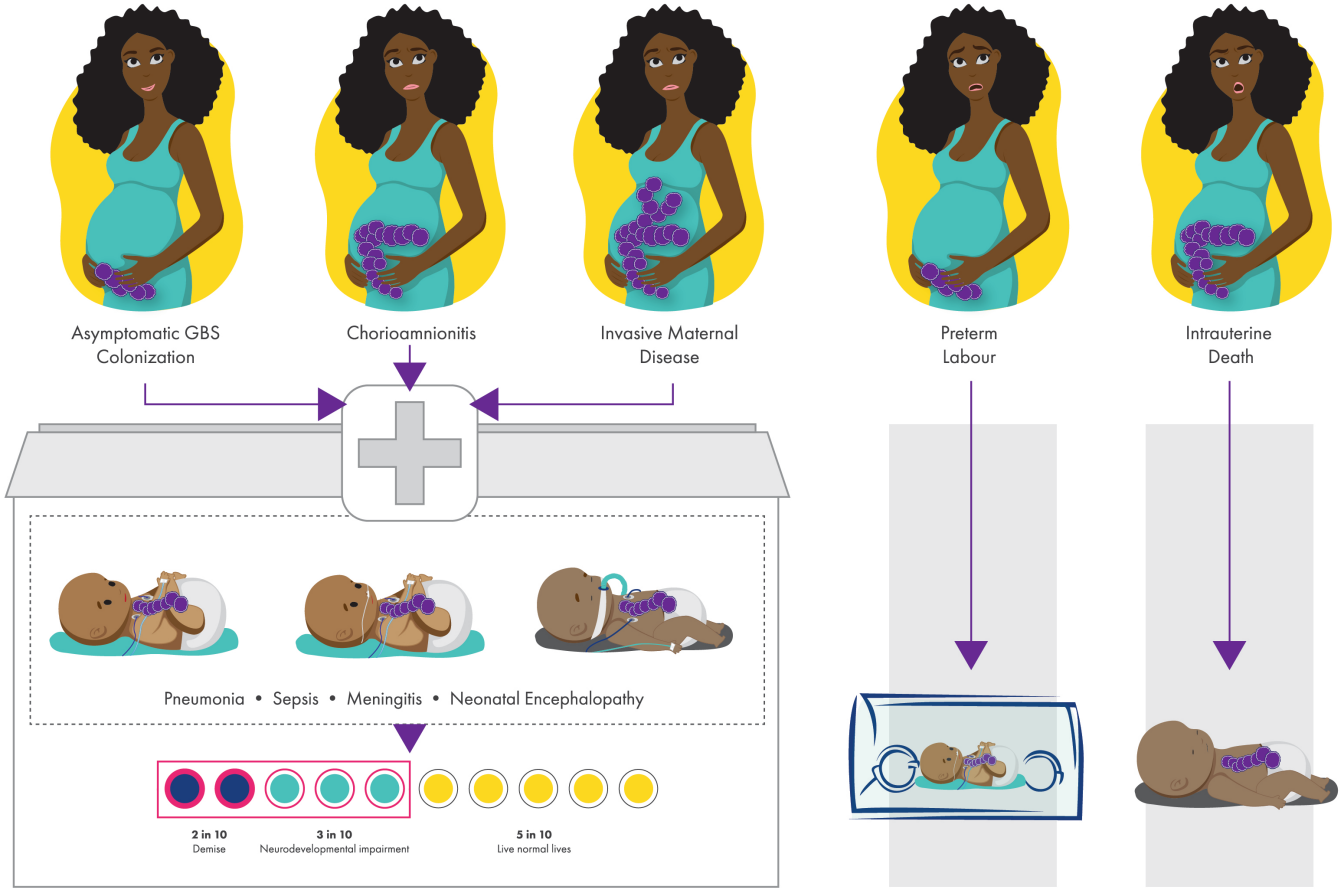
Perinatal and birth outcomes of newborns with invasive group B streptococcal (GBS) disease. GBS colonisation can lead to invasive early-onset neonatal disease (pneumonia, sepsis, meningitis, neonatal encephalopathy), and can also result in maternal infection (chorioamnionitis and invasive maternal disease), preterm birth and stillbirth. In sub-Saharan Africa, 23% will demise and 38% of survivors from low- and middle-income countries have neurodevelopmental impairment.

GBS colonization is usually asymptomatic, and maternal GBS colonization status is unknown to most African pregnant women as this is not routinely tested for in pregnancy. During the prenatal or perinatal period, ascending GBS into amniotic cavity can adversely affect the pregnant women resulting in chorioamnionitis or invasive maternal disease (15). For the foetus or newborn, the possible outcomes are EOD (pneumonia, sepsis, meningitis and/ or neonatal encephalopathy), stillbirth, or prematurity (11–13, 19, 20, 24) (Figure 1).

## Current preventative strategies

Currently, prevention of invasive GBS EOD is dependant on the availability of intravenous antibiotics that can be administered to a pregnant women at least 4 h before delivery by adequately trained health care workers (35). There are two screening approaches (microbiological or clinical risk-based) to determine which pregnant women should get IAP. Many high-income countries use the microbiological screening approach in which pregnant women have a vaginal-rectal swab in the 36 or 37th week of gestation, and are offered IAP if they are GBS colonized (36). This strategy resulted in a 80% reduction in EOD in the United States from the early 1990’s to 2010 (37). The risk-based approach targets pregnant women that have selected clinical risk factors (premature labour, PROM, maternal fever or previous child with GBS). Both strategies have limitations; microbiological screening is insensitive and colonisation status changes during pregnancy. Clinical risk factors are non-specific, and only identified with close monitoring and comprehensive assessments, otherwise sensitivity is low. Microbiological screening also requires processing of specimens, logistical support and timely feedback of results. Microbiolgical screening is likely more effective at reducing EOD than risk-based approaches (38), but implementation challenges make either strategy challenging to implement in resource limited African settings. In African countries, IAP is not provided due to an absence of microbiological screening, and (i) a large proportion of home deliveries, (ii) no intravenous treatment available at primary health care facilities where a large proportion of women deliver, (iii) inability to administer antibiotics 4 h before delivery because of late presentations to labour ward, or staff shortages that delay medical interventions, and (iv) failure to recognise pregnant women with risk factors when they are present (39). Importantly, the risk of EOD to a neonate born to GBS colonized pregnant women is 1.1% with no IAP compared to 0.3% where IAP coverage is high (34).

Point of care / real-time molecular PCR testing in labour wards with IAP to colonized women has also been associated with a reduction in EOD (40). The sensitivity and specificity of PCR testing to detect GBS colonization in pregnant women has been reported as 93 and 97%, respectively (41). The advantages of these point of care testing would be a non-reliance on laboratory service and the immendiate availability of results to the midwife / obstetrician, but this will need to be offset by the high cost of these tests.

Recent studies have investigated the role of probiotics, garlic and zinc in the prevention of GBS colization but results are inconclusive (42–44).

## Maternal GBS vaccination

Maternal vaccination is now recognized as the optimal strategy to reduce disease in pregnant women, and foetus or young infant. There is considerable precedent for vaccines in pregnancy providing protection at the most critical time after birth, and WHO has recommended tetanus, pertussis, influenza and Covid-19 vaccinations during pregnancy (45). Vaccinating pregnant women against pertussis and influenza has been shown reduce disease in infants of vaccinated mothers, and has been associated with a 75% reduction in stillbirths and a 30% reduction in preterm birth (46–48). Other vaccines such as those for Cholera and Typhoid are recommended in specific situations, and a number of maternal vaccines are currently under development (45).

Vaccination of pregnant women to prevent invasive GBS disease in infancy is an important, pragmatic, future preventative strategy for Africa and other low- and middle-income countries (LMICs). The WHO has provided a comprehensive value assessment for a GBS vaccine (49–51). A GBS vaccine would also likely prevent stillbirths and LOD (51), and may reduce GBS-associated preterm birth. A maternal GBS vaccine would need to be safe, effective, affordable, and accepted by policymakers as important, with demand from pregnant women and their partners. Studies from high-income countries suggest there is likely to be demand, but studies from countries in Africa are needed; in a study from the United Kingdom, after receiving information about GBS, two thirds of women reported that they would consider taking a GBS vaccine during pregnancy, underpinning the need for interventions to support uptake (52).

In terms of health impact and cost-effectiveness, a maternal GBS vaccine with 80% efficacy could prevent, worldwide, 127,000 (uncertainty range (UR): 63,300 – 248,000) EOD cases, 87,300 (UR: 38,100 – 209,000) LOD cases, 31,100 (UR: 14,400 – 66,400) deaths, 17,900 (UR: 6,380 – 49,900) cases of moderate and severe NDI, 185,000 (UR: 13,500 – 407,000) preterm births, and 23,000 (UR: 10,000 – 56,400) stillbirths per year (53). The effect will be greatest in sub-Saharan where two-fifths of the above will be prevented by vaccinating a fifth of women (53). A GBS vaccine is likely to be cost effective in most settings, including African countries (54–56). Asssuming the cost of the vaccine was $50, $15 and $3.50 in high, upper-middle, and in low/lower-middle-income countries, respectively, a single dose of the vaccine would cost $1.7 billion but save $385 million in healthcare costs worldwide (53).

The mechanism of protection against invasive disease through maternal vaccination is from an increase in type-specific antibody levels in the vaccinated pregnant women that can be transplacentally transferred to the foetus over the weeks (most tranfer occurs in the last trimester) until delivery. The transplacental transfer of type-specific maternal IgG antibodies (IgG1 > IgG4 > IgG3 > IgG2) is an active process from the maternal to foetal circulation using the neonatal Fc receptor (FcRn) (45). Protein based vaccines induce mostly IgG1 and IgG3 responses, whereas capsular polysaccharide vaccines induce IgG2. The rate of IgG transfer is negatively affected by infections such as malaria and HIV, which are common in some areas of African countries. For a maternal GBS vaccine to be effective, the vaccine would need to be: (i) immunogenic and elicit functional responses, and (ii) administered at the appropriate gestational age for optimal concentrations of IgG to be transported across the placenta to the foetus (this is dependant on IgG subclass, transfer rate and gestational time to birth). Importantly, a vaccine to prevent invasive GBS disease can reduce the emergence of antimicrobial resistance; recent data from the US shows a low but increasing proportion of isolates with reduced beta-lactam susceptibility (57).

### Vaccine licensure pathway and current landscape

In 1976, Baker and Kasper reported that low levels of maternal antibody titer was associated with invasive GBS disease (58). Both the GBS capsular polysaccharide (CPS) and many surface proteins have antigenic properties. Many studies have reported the association between higher levels of natural acquired maternal antibodies against GBS CPS and surface protein, and decreased acquisition of GBS colonization or a decreased risk of invasive GBS disease (59–64). Using Bayesian analysis, varying thresholds (“correlates of protection”) have been proposed (62). A maternal IgG antibody concentration of ≥1 μg/mL for serotypes Ia, III and V was proposed as a threshold of protection amongst pregnant women in the US by Baker and colleagues in 2014 (65). This same threshold reduced the risk of disease by 80% for serotypes Ia and III from eight European countries (66). In South Africa, however, a higher threshold of protection was proposed (≥2.3 μg/mL and ≥ 3.4 μg/mL for serotypes Ia and III was associated with a 90% risk reduction in disease) (63). Importantly, benchmarking a correlate of protection to infant antibody levels, and supplementing that with maternal levels and transplcental tranfer ratios may be necessary because of the unpredictability of transplacental transfer. An infant antibody level between 1 and 3 μg/mL protected 90% of infants from invasive GBS disease caused by serotype Ia and III in South Africa (63, 67). Determination of single protective threshold for all GBS serotypes would be ideal.

There have been a number of monovalent, bivalent, trivalent (Ia, Ib, III) and hexavalent (Ia, Ib, II, III, IV and V) CPS phase I and II vaccine trials over the past few decades, some of which were undertaken in pregnant women (68–70). These trials showed higher IgG antibody reponses with CPS protein conjugate vaccines compared to CPS only vaccines. Investigators also explored using the tetanus toxoid versus CRM197, a non-toxic mutant of diphtheria toxin, as carrier protein – findings were similar (68). Furthermore, CPS protein conjugate vaccines induced peak responses 4–8 weeks after vaccination and began to wane 6 months after vaccination (68). The first GBS vaccine trial undertaken in pregnant women was conducted in the United States in 1988 using an unconjugated monovalent serotype III CPS vaccine, and then followed this up with a serotype-III CPS protein conjugate vaccine in 2003 (71, 72). Subsequently, phase Ib/II trivalent CPS protein conjugate vaccine trials have explored safety, different dosing schedules, a second dose, and immunogenicity in HIV-infected compared to uninfected pregnant women (73–77). These GBS CPS conjugate vaccines were safe in pregnancy. In South Africa, a trivalent GBS vaccine administered to pregnant women showed higher geometric mean concentrations (GMCs) than placebo for serotypes Ia, Ib and III, and a significant difference was noted with higher vaccine doses for serotype Ia, but not for Ib and III. Cord-maternal antibody ratios were 49–79% (74). Similarly, infants of vaccinated mothers had higher serotype-specific GMCs than placebo, and serotypes Ia and III levels decreased to 26–35% from birth to 3 months of age (78). Therefore, a GBS polysaccharide conjugate vaccine is likely to protect young infants from invasive GBS disease. Additionally, a phase II, GBS serotype III conjugate vaccine administered to nonpregnant women was 36–43% effective at reducing first acquisition of GBS vaginal/rectal colonization (79). Furthermore, the vaccine is likely to increase sIgA breastmilk antibody concentrations; serotype-specific natural sIgA antibody was independently associated with protection against LOD (80).

Given the serotype diversity globally and the increased prevalence of serotype IV and V in some regions, a Pfizer hexavalent (Ia, Ib, II, III, IV, and V; referred to as GBS6) vaccine is undergoing clinical trials. The phase I/II, placebo-controlled, dose-escalation trial in 365 healthy men and non-pregnant women aged 18–49 years who received GBS6 (5 μg, 10 μg, 20 μg) with aluminium phosphate (AlPO4) or placebo reported adverse events in 23–48% of participants depending on dosing and inclusion of AlPO4 (81). None of three serious adverse events reported during the study were considered to be related to the vaccine. Serotype-specific IgG GMCs peaked at 2 weeks after vaccination and remained higher (10 to 56-fold rise) than placebo at six-months. Most (75%) vaccinated participants had an antibody concentration ≥ 1 μg/mL for serotypes Ia, II, III, IV, and V at 1 month after vaccination. A threshold of ≥1 μg/mL for serotype Ib was present in 40–57% of participants. GBS6 is being evaluated in an ongoing phase II, placebo-controlled, study in pregnant women assessing the safety, tolerability and immunogenicity (NCT03765073). Another hexavalent vaccine candidate developed by Inventprise is in the preclinical phase.

There are few reasons for the considerable delays in phase III trials of GBS conjugate vaccines. Firstly, the burden of invasive disease needed to be better established. A geographic diversity in the GBS capsular serotypes causing disease were observed, and vaccines were increased from trivalent to hexavalent, aiming to cover 98% of serotypes causing disease in young infants (16). Secondly, the full value proposition of vaccination that extends beyond invasive neonatal disease needed to be defined. This includes the potential impact of vaccination on all-cause neonatal sepsis, neonatal mortality and stillbirths. Thirdly, licensure of the vaccine through conventional phase III clinical trials with clinical outcomes of efficacy would mean that a sample of 30–180,000 pregnant women would be required in a setting with a high burden of disease (82, 83). Therefore, licensure of a GBS vaccine would be facilitated, if acceptable to regulators, through benchmarking the efficacy endpoint on a threshold of protection that is required to protect against invasive infant GBS disease, and following that with phase IV clinical effectiveness studies (83). This approach has been previously explored for meningocococcal and pneumococcal vaccines (84, 85).

Protein based vaccines that are highly conserved and provide a broad spectrum of protection against most serotype-causing disease strains are also under development. Amongst the leading candidates are antigens tartgetted at the Alpha family (Alp 1–4, Alpha C and Rib) of surface proteins. A protein based GBS vaccine that consists of the N-terminal domains of AlphaC and Rib (GBS-NN) is immunogenic and safe in non-pregnant adults (86). Similarly, a second generation vaccine (AlpN) that includes Rib, Alpha C, Alp1, Alp2 and Alp3 has been shown to be immunogenic and safe in non-pregnant adults (87). Currently, a phase 2 (NCT05154578) study evaluating the immunogenicity and safety of the AlpN vaccine in pregnant women is underway in South Africa, Denmark, Uganda and United Kingdom. Thresholds for protection have also been proposed for some of these surface protein vaccine candidates (64).

The GBS vaccine pipeline has been recently described (70, 88); leading candidates are the Pfizer hexavalent CPS protein conjugate vaccine and the Minervax AlpN protein-based vaccine. Monovalent, bivalent or trivalent investigational CPS protein conjugate vaccine that reached phase II evaluation have been suspended. Notably, few CPS protein conjugate and protein-based vaccine are in pre-clinical trials.

### Considerations for GBS vaccination in African populations

There are specific considerations for vaccines in African populations. The prevalence of HIV amongst pregnant women in some sub-Saharan African countries is high. In South Africa, the antenatal HIV prevalence rate has remained at 30% over the last two decades (89). Despite advancements in the prevention of mother to child programmes of HIV, HIV-infected pregnant women have an increased risk of having a baby with invasive GBS disease (90, 91). HIV-exposed neonates have lower serotype-specific and surface protein antibody levels at birth compared to HIV-unexposed neonates (92, 93). This may in part be from lower naturally acquired antibody levels in HIV-infected pregnant women and secondly, from inefficient transplacental transfer of antibody (92, 93). Notably, in Malawi and South Africa, a 5 μg trivalent (serotypes Ia, Ib, and III) GBS vaccine was found to be less immunogenic in HIV-infected compared to HIV-uninfected pregnant women (75). Therefore, higher doses or alternative dosing schedules will need to be considered in HIV-infected pregnant women to optimise antibody responses and tranplacental transfer.

The second concern in African populations is that malaria is endemic to many regions. Similar to HIV, malaria may affect the transplacental transfer of antibody from the pregnant women to the foetus, this needs to be evaluated in the context of a GBS vaccine (94).

Thirdly, prematurity is prevalent in Africa, and vaccinated pregnant women need an optimal gestational period after vaccination to achieve adequate antibody transfer to the foetus. Approximately half of maternal antibody levels are present in the foetus at the beginning of the third trimester (95). Therefore, early preterm neonates will be at increased risk of disease. This is a major challenge for most African countries where IAP for pregnant women presenting in preterm labour in not readily available.

Lastly, some of the highest burden of GBS disease is in sub-Saharan Africa and therefore ethical considerations to undertaking clinical trials in these settings need to consider implementation and sustainability of the vaccine after licensure (96).

### Post GBS vaccine licensure

If licensure is based on a correlate of protection, this will need to be followed by vaccine effectiveness studies, vaccine probe studies or national / regional surveillance programmes to measure the impact of vaccination on culture-confirmed GBS disease and stillbirths, all-cause sepsis, all-cause meningitis, all neonatal deaths and all stillbirths, and the effect of the vaccine on preterm labour. In addition, safety monitoring; particularly foetal and birth outcomes will need to continue. For a successful GBS vaccine, health-care worker (obstetrician/midwife) engagement is crucial. To overcome vaccine hesitancy, public engagement and awareness programmes are required by governments and policymakers. Importantly, as more maternal vaccines are recommended, an antenatal extended programme of immunisation (EPI) for pregnant women needs to be developed and understanding vaccine interactions and timing during pregnancy needs to be clearly defined.

## Conclusion

The burden of invasive GBS disease, the commonest cause of EOD in neonates, is high in Africa, where IAP preventative strategies are not feasible, although data gaps remain. However, after many decades of GBS vaccine development, maternal GBS vaccines are moving into late stage clinical trials. If these vaccine candidates are successful and a GBS vaccine is licensed based on a correlate of protection, studies of effectiveness will need to follow to assess the burden of GBS vaccine preventable disease. These studies should be complemented by routine clinical surveillance (97). This will require sentinel surveillance sites with standardized laboratory methods and monitoring systems to investigate neonates with signs of infection and confirmation of GBS, similar to those used in the estimation of pneumococcal and rotavirus vaccination effectiveness (98). These WHO surveillance sites were designed to measure baseline burden of disease or cost-effectiveness, estimate VE after vaccine introduction, monitor trends in serotype/ genotype distribution and serotype replacement after vaccine introduction, and detect other infectious diseases. Such sites exist in African countries, but sustainability is challenging.

## Data availability statement

The original contributions presented in the study are included in the article/supplementary material, further inquiries can be directed to the corresponding author.

## Author contributions

ZD wrote the first draft. AS, VB, and GK provided critical review. All authors contributed to the article and approved the submitted version.

## Conflict of interest

AS is employed by the Bill and Melinda Gates Foundation.

The remaining authors declare that the research was conducted in the absence of any commercial or financial relationships that could be construed as a potential conflict of interest.

## Publisher’s note

All claims expressed in this article are solely those of the authors and do not necessarily represent those of their affiliated organizations, or those of the publisher, the editors and the reviewers. Any product that may be evaluated in this article, or claim that may be made by its manufacturer, is not guaranteed or endorsed by the publisher.
